# A simple and cost-effective method for screening of CRISPR/Cas9-induced homozygous/biallelic mutants

**DOI:** 10.1186/s13007-018-0305-8

**Published:** 2018-05-29

**Authors:** Jinggong Guo, Kun Li, Lifeng Jin, Rui Xu, Kaiting Miao, Fengbo Yang, Chaoya Qi, Lin Zhang, Jose R. Botella, Ran Wang, Yuchen Miao

**Affiliations:** 1 State Key Laboratory of Cotton Biology, Department of Biology, Institute of Plant Stress Biology, Henan University, 85 Minglun Street, Kaifeng, 475001 China; 2 Zhengzhou Tabacco Research Institute of CNTC, No. 2 Fengyang Street, Zhengzhou, 450001 Henan China; 3 School of Life Science, Southwest University, No. 1, Tiansheng Road, Beibei, 400715 Chongqing China; 4 College of Tobacco Science, Henan Agricultural University, No.63 Agriculture Road, Zhengzhou, 450002 Henan China; 5 School of Agriculture and Food Sciences, University of Queensland, Brisbane, QLD Australia

**Keywords:** CRISPR/Cas9, Genome editing, Tobacco, *Arabidopsis thaliana*, PCR, Mutant screening

## Abstract

**Background:**

The CRISPR/Cas9 system is being used for genome editing purposes by many research groups in multiple plant species. Traditional sequencing methods to identify homozygous mutants are time-consuming, laborious and expensive.

**Results:**

We have developed a method to screen CRISPR/Cas9-induced mutants through Mutation Sites Based Specific Primers Polymerase Chain Reaction (MSBSP-PCR). The MSBSP-PCR method was successfully used to identify homozygous/biallelic mutants in *Nicotiana tabacum* and *Arabidopsis thaliana*, and we speculate that it can be used for the identification of CRISPR/Cas9-induced mutants in other plant species. Compared to traditional sequencing methods, MSBSP-PCR is simpler, faster and cheaper.

**Conclusions:**

The MSBSP-PCR method is simple to implement and can save time and cost in the screening of CRISPR/Cas9-induced homozygous/biallelic mutants.

**Electronic supplementary material:**

The online version of this article (10.1186/s13007-018-0305-8) contains supplementary material, which is available to authorized users.

## Background

The availability of genetic mutants is essential for functional studies as well as to determine genetic relationships such as epistatic associations in genetic pathways. The discovery of sequence-specific nucleases (SSNs) provided the tools for genome editing, allowing the introduction of mutations in specific chromosomal loci and conveyed the potential to revolutionize biological and medical research. The most widely used SSNs include zinc-finger nucleases (ZFNs) [1, 2], transcription activator-like effector nucleases (TALENs) [3] and Clustered Regularly Interspaced Short Palindromic Repeats (CRISPR)/CRISPR-associated protein 9 (Cas9) [4–6]. All three types of nucleases have been successfully used to generate mutations by producing targeted DNA double-strand breaks (DSBs), which are repaired by either the error-prone non-homologous end joining (NHEJ) repair pathway or the high-fidelity homologous recombination pathway. In order to perform their function, ZFNs and TALENs contain arrays of peptide-based DNA-binding domains fused to the nonspecific DNA cleavage domain from the restriction enzyme *Fok*I. The amino acid sequences of the zinc-finger and TALE arrays can be designed to bind almost any target DNA sequence with high specificity [7–11]. However, the protein-DNA interactions in ZFNs and TALENs are quite complex and newly designed proteins need to be experimentally validated [12–14].

In addition, construction of the ZFN and TALEN vectors is technically demanding, limiting their widespread adoption by the scientific community. In contrast, DNA targeting in the CRISPR-Cas9 system is provided by a relatively short RNA sequence, the single-guide RNA (sgRNA), which directs the Cas9 protein to the correct chromosomal position by base complementary in order to generate DSBs. The target sequence is approximately 20 base pairs (bp) long and is located directly upstream of a protospacer-adjacent motif (PAM). Although the CRISPR/Cas9 system is somewhat limited by the requirement of a suitable PAM next to the target sequence, its technical simplicity compared to the complex protein design and engineering tasks associated with ZFNs and TALENs has made it the tool of choice for genome editing [15]. In a very short period of time, the CRISPR-Cas9 system has been successfully used to generate precise mutations in multiple crops including cotton, rice, wheat and potato [16–21].

Most mutations generated by the CRISPR/Cas9 system are either insertions or deletions usually located close to the DSB site that occur 3 bp upstream of the PAM [15]. A common approach to elucidate the nature of the mutations generated by CRISPR is to amplify a fragment of the targeted-gene by polymerase chain reaction (PCR) and sequence the PCR amplicons. The mutagenesis efficiency of the CRISPR system varies according to the plant species and the targeted sequences, therefore efficient screening strategies are paramount to identify individuals carrying mutations in the T_0_ or subsequent generations. A number of different approaches have been developed in order to screen for mutants including PCR/restriction enzyme (RE) assay, T7 endonuclease I (T7EI) assay, surveyor nuclease assay, polyacrylamide gel electrophoresis (PAGE)-based methods, high-resolution melting (HRM) analysis-based assays, fluorescent PCR-capillary gel electrophoresis methods and annealing at critical temperature PCR (ACT-PCR) assays [15, 22–28]. All of these approaches have been successfully used but each has a number of limitations including the amount of time and labour required, cost, low detection specificity, expensive equipment requirements or in the case of PAGE-based methods, the inability to identify individuals with homozygous mutations.

Here, we describe a simple, reliable and inexpensive method to screen for the presence of CRISPR-Cas9-induced mutations. The method, named Mutation Sites Based Specific Primers PCR (MSBSP-PCR) uses optimized parameters for tandem PCR-based analysis. The method has been validated by screening CRISPR/Cas9-induced mutants in T_0_ transgenic tobacco plants and T_1_ transgenic tobacco and *Arabidopsis* plants.

## Methods

### Plant materials

Seeds of *Arabidopsis thaliana* (ecotype Columbia-0) were grown on half-strength Murashige and Skoog media (MS) [29] supplemented with 0.6% (w/v) agarose for 7 d in controlled environmental conditions of 21 °C and a 16-h-light/8-h-dark photoperiod, with the light intensity of 150 μmol m^−2^ s^−1^. The seedlings were transferred to soil for 15–20 d for DNA extraction in the same environmental conditions, and about 0.03–0.05 g rosette leaves were used for DNA extraction.

Seeds of *Nicotiana tabacum* L. (K326) were obtained from the seed stocks bank in our laboratory (National Tobacco Gene Research Center, Zhengzhou, China). Seeds were surface-sterilized with bleach containing 30% sodium hypochlorite for 30 min and grown on half-strength MS media [29] supplemented with 0.6% agarose for 7 d in controlled environmental conditions of 25 °C and a 16-h-light/8-h-dark photoperiod, with the light intensity of 150 μmol m^−2^ s^−1^. The seedlings were transferred to soil for 15-20 d for DNA extraction with 0.05–0.1 g true leaves. The tobacco plants were grown in a greenhouse maintaining day/night temperature at 28/23 °C and 16-h-light/8-h-dark photoperiod.

### *Agrobacterium*-mediated tobacco transformation

*Agrobacterium*-mediated tobacco transformation was performed as previously described [30].

### CRISPR/Cas9

The CRISPR/Cas9 vectors were provided by Prof. Qijun Chen, from China Agricultural University [31]. Sequences of each tobacco gene, the PAM sites and CRISPR/Cas9-induced mutants or synthesized templates used in all experiments are listed in Additional file 1.

### PCR

Tobacco genomic DNA (gDNA) was extracted using a Plant DNA Isolation Kit following manufacturer’s instructions (Foregene, China). gDNA concentration was measured using a NANoDROP 2000c spectrophotometer (Thermo scientific). If the PCR product was used for sequencing analysis, PCR amplification reactions were performed using Phanta^®^ Max Super-Fidelity DNA Polymerase (Vazyme) in a final volume of 20 μL, containing 10 μL of 2 × Phanta Max buffer, 1.6 μL of 2.5 mM dNTP Mix, 1 μL of forward primer (10 μM), 1 μL of reverse primer (10 μM), 40 ng of gDNA, and 0.4 μL of DNA polymerase (1 U/μL). If the PCR product was used for agarose electrophoretic analysis, PCR amplification were conducted in final volumes of 20 μL, using Taq Master Mix (novoprotein), which contained 10 μL of 2 × Master Mix, 1 μL of forward primer or target primer (10 μM), 1 μL of reverse primer (10 μM) and 40 ng of gDNA.

PCR amplifications were performed using the following parameters: 95 °C for 5 min; 16–30 cycles (suitable cycles were chosen for each gene) of 95 °C for 30 s, 56–65 °C (proper annealing temperature was chosen for gene) for 30 s, and 72 °C for 50 s, with a final extension step of 72 °C for 5 min. In general, 30 cycles are suitable for most genes in the first PCR (with two external primers) with 40 ng gDNA as templates. The products of the first PCR are analyzed by gel electrophoresis, and same amount of the products (1–2 ng) can be used as templates of the second PCR, with a target primer (T-primer) expanding the sites of the expected CRISPR-Cas9-induced mutations and one of the external primers used in the initial amplification. All primers used in this study are listed in Additional file 2.

## Results

### Principles and schematic overview of MSBSP-PCR

**Fig. 1:**
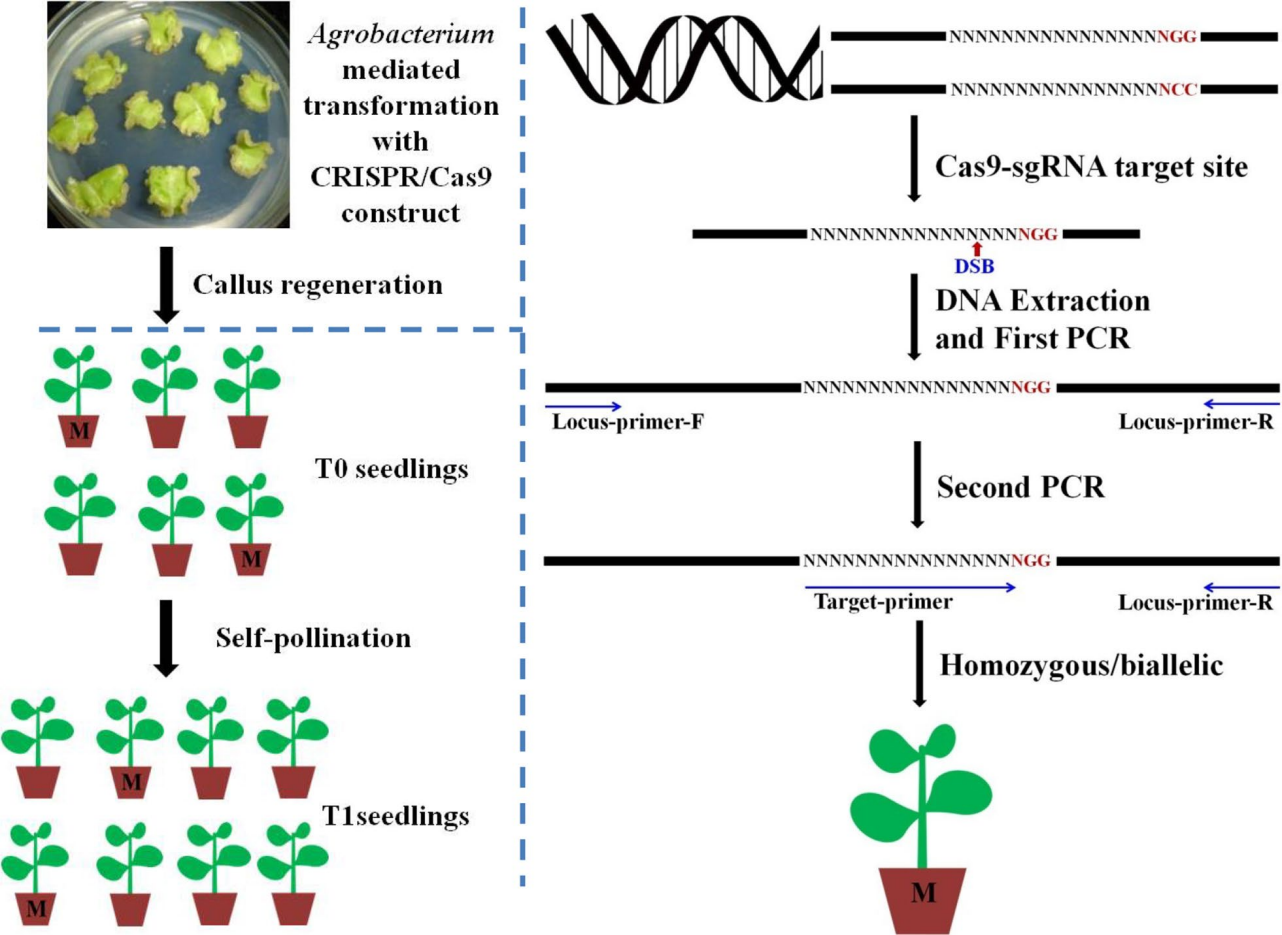
Schematic overview of the Mutation Sites Based Specific Primers PCR (MSBSP-PCR) method to identify CRISPR/Cas9-induced mutants in tobacco. CRISPR/Cas9 constructs were transferred to tobacco plants using *Agrobacterium* mediated transformation. Genomic DNA from either T_0_ or T_1_ plants was purified and subjected to a first PCR amplification using Locus-primer-F (forward primer) and Locus-primer-R (reverse primer). The products of the primary amplification were then used in a secondary PCR using a Target-primer and the Locus-primer-R. The target primer is a mutation site-specific primer and expands the recognition site for the sgRNA. WT plants and heterozygous mutants will produce an amplification product in the secondary PCR while homozygous/biallelic mutants will not show any amplification

The MSBSP-PCR method was initially developed to identify CRISPR/Cas9-induced mutants in transgenic tobacco lines for our research. Figure 1 shows the schematic overview of mutant establishment and MSBSP-PCR screening in tobacco. *Agrobacterium* mediated transformation is used to produce T_0_ transgenic lines containing CRISPR/Cas9 cassettes targeting a specific chromosomal locus. Individual T_0_ plants undergo screening to identify those containing mutations by performing two sequential rounds of PCR amplification. The first PCR reaction is performed using genomic DNA from the T_0_ transgenic plants with two external primers designed to anneal 200-300 bp upstream and downstream from the PAM site respectively (Locus-primer-F/Locus-primer-R, Fig. 1). The amplified PCR product is expected to contain the targeted locus and any mutations caused by the CRISPR/Cas9 expression cassette and should be analysed by gel electrophoresis. The product of the first amplification (1–2 ng) is then subjected to a second PCR amplification using a target primer (T-primer) expanding the site of the expected CRISPR-induced mutation and one of the external primers used in the initial amplification. The PCR parameters for this second amplification are critical for the success of the method and need to be optimized.

CRISPR/Cas9 produces DSBs 3 bp upstream of the PAM that is subsequently repaired by the NHEJ cellular machinery. The error prone nature of the NHEJ will typically introduce mutations at the repair site, with the most frequent ones being small (1-3 bp) deletions or insertions [15]. A primer designed to anneal at the recognition site of the sgRNA will have an imperfect match in those cases where a mutation has taken place and under stringent annealing conditions will fail to produce an amplification product. It is therefore essential to determine the ideal melting temperature (Tm) for the second PCR in a way that it will allow amplification of WT templates but will fail to amplify mutated templates.

**Fig. 2:**
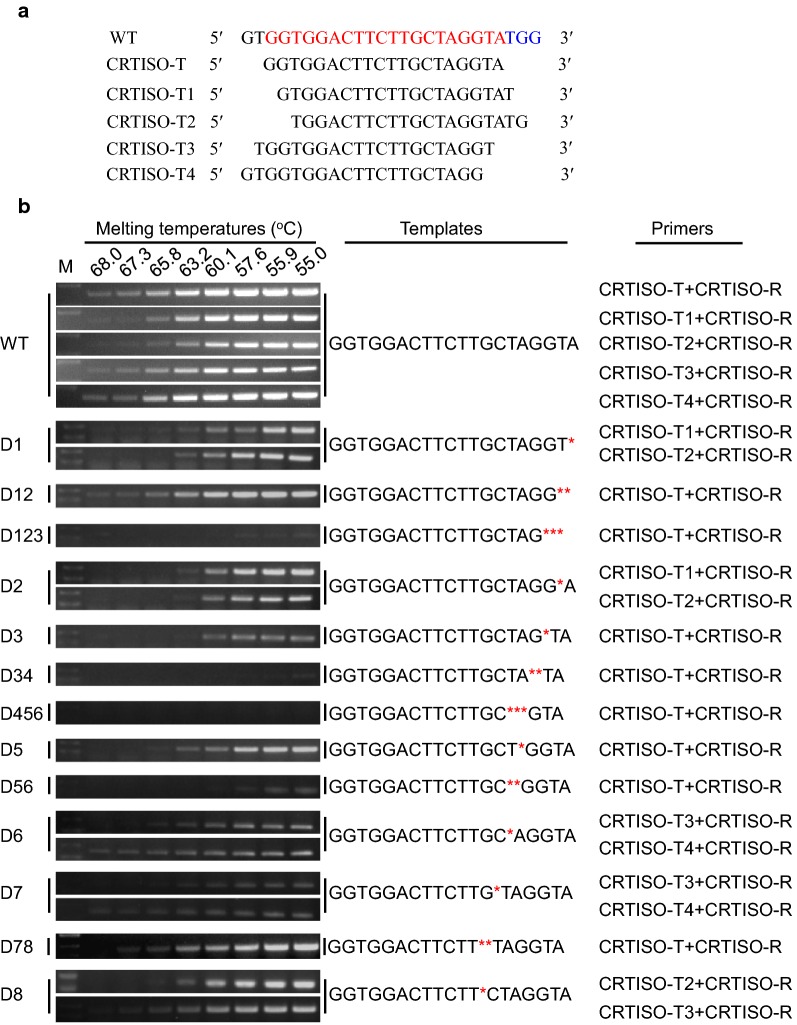
PCR amplification of some synthesized mutated templates with different primer combinations. **a** WT sequence and PCR primers used in the amplification reactions. The CRISPR/Cas9 target sequence (red) and PAM (blue) are shown in the WT sequence. **b** Gel electrophoresis was used to analyze the PCR products for different templates and primer combinations. Red asterisks denote the position of the nucleotide deletions in the templates

To verify the feasibility of MSBSP-PCR in the identification of CRISPR/Cas9-induced mutations and provide clues for the design of suitable target-primers we used the *Nicotiana tabacum* prolycopene isomerase 1 gene: *NtCRTISO* (GenBank accession number XM_016608861.1). Multiple *NtCRTISO* primers (Fig. 2a) and templates containing different mutations (Fig. 2b) in the sgRNA target site were synthesized. The nature of the mutations were small deletions (1–3 bp) in the vicinity of the PAM as it has been reported that these types of deletions are the most common ones induced by the CRISPR/Cas9 system in plants [15]. Five different target-primers were designed around the sgRNA recognition site and the optimal annealing temperature for the MSBSP-PCR reaction established by performing PCR reactions over a temperature gradient (Tm = 55–68  °C, 28 cycles, Fig. 2b). When PCR was performed with the WT *NtCRTISO* template and the CRTISO-T & CRTISO-R primer combination, amplification products were observed at all Tms although the intensity of the bands decreased with increasing Tm. As expected, PCR reactions using the different mutant templates and primer combinations produced a variety of results. While many of the primer combinations produced an amplicon at the minimum tested temperature (55 °C) using mutant templates (Fig. 2b and Additional file 3: Fig. 1), some mutation/primer combinations completely failed to amplify any DNA (see D123, D34, and D456 with CRTISO-T/CRTISO-R primers in Additional file 4: Fig. 2). In general, very weak or no amplicon bands were observed at annealing temperatures above 60 °C, some primer combinations showed little products even at annealing temperatures above 57 °C. The CRTISO-T/CRTISO-R primer combination proved to be the most efficient in recognizing the presence of mutations with the CRTISO-T primer being positioned exactly in the target site for the sgRNA. The design of suitable primers and determination of the optimal annealing temperature is highly dependent on the nature of the mutation generated by the CRISPR/Cas9 system and some additional optimization might be needed in some cases. To determine the optimal primers and annealing temperatures in problematic cases, cloning of the amplicon generated in the first PCR (using CRTISO-T and CRTISO-R primers) into TA-based plasmids might be needed followed by sequencing of a number of recombinant bacterial clones to confirm the nature of the mutation. Bacterial clones containing a mutation can then be used to optimize the PCR parameters in order to distinguish between WT and mutated templates.

In conclusion, for the first PCR of MSBSP-PCR, annealing temperature is determined by the locus-primer-F and locus-primer-R Tms and the cycle number should be experimentally determined by performing an amplification reaction and taking 3–5 μL samples at 20, 25, 30 and 35 cycles, analyzing them in an electrophoresis gel and looking for the minimum amount of cycles that yields a single and clear band. To determine the parameters for the secondary PCR, Tms for the target-primer and locus-primer-R are calculated and an optimization PCR performed using product of the first PCR (WT genomic DNA as template) and an annealing temperature gradient with an upper limit of 7–12 °C above the optimal temperature. The optimal cycle number is determined as explained above, by analysing samples by electrophoresis at different cycle numbers, looking for the presence of a clear and single amplicon band.

### Identification of CRISPR/Cas9-induced mutants in tobacco and *Arabidopsis* by MSBSP-PCR

**Fig. 3:**
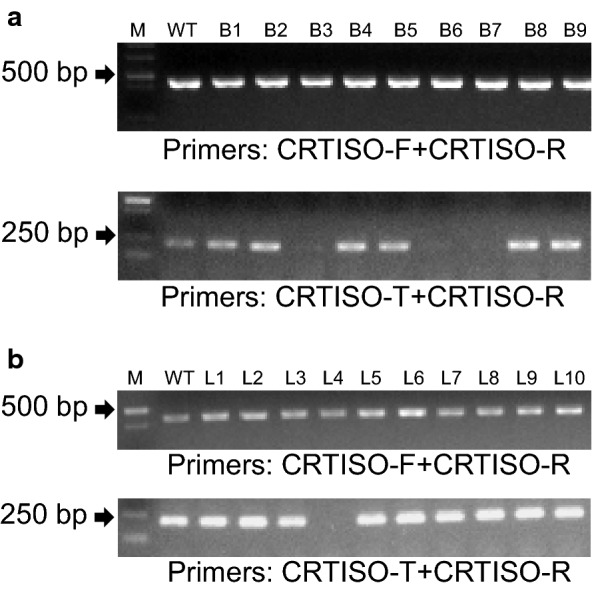
Identification of CRISPR/Cas9-induced *crtiso* mutants in tobacco by MSBSP-PCR. **a** Genomic DNA from T_0_ plants was used for PCR amplification using primers CRTISO-F and CRTISO-R. The amplification products (with gDNA from a putative T_0_ transgenic tobacco line as template) were cloned into a TA-vector and recombinant bacteria screened for the presence of mutations at the target site. The absence of amplification product when using the target specific primer CRTISO-T and CRTISO-R denotes the existence of a mutation (bacterium B3, B6 and B7 in lower panel). As a positive control bacterial clones were also amplified with CRTISO-F and CRTISO-R (upper panel). **b** Genomic DNA from T_0_ tobacco transgenic lines was amplified by PCR with primers CRTISO-F and CRTISO-R (upper panel) and the amplification products subjected to a second PCR using CRTISO-T and CRTISO-R primers (lower panel). The absence of an amplification product in the second PCR suggests that the transgenic line is homozygous/biallelic (lower panel lane L4)

To verify the usefulness of the MSBSP-PCR method in real experimental conditions, a CRISPR/Cas9 construct targeting the *NtCRTISO* gene (Fig. 2) was cloned in a binary vector [31], and 39 putative T_0_ transgenic tobacco lines produced by *Agrobacterium*-mediated transformation. For each T_0_ plant, a fragment containing the targeted region in *NtCRTISO* was amplified from purified genomic DNA using the CRTISO-F/CRTISO-R primers (Tm = 53 °C, 30 cycles) (Additional file 5: Fig. 3A). As a preliminary step to validate the parameters for the secondary PCR reaction, the primary amplification products from each putative T_0_ transgenic tobacco line were cloned into a TA cloning vector and a number of recombinant clones used as templates to perform PCR reactions using primers CRTISO-T/CRTISO-R (Tm = 62 °C, 20 cycles) to determine the presence of mutations. As shown in Fig. 3, with gDNA from a putative T_0_ transgenic tobacco line (line 29 in Additional file 5: Fig. 3) as template, the amplification products were cloned into a TA-vector and recombinant bacteria (verified by PCR with primers of CRTISO-F/CRTISO-R, Fig. 3a, upper panel) screened for the presence of mutations (Fig. 3a, bottom panel), the absence of amplification products in bacterium B3, B6 and B7 indicated the presence of mutations and where further confirmed by sequencing of the clones (Additional file 6: Fig. 4). PCR amplifications using primers CRTISO-F/CRTISO-R (Tm = 53°C, 30 cycles) were also performed as positive controls (Fig. 3a, top panel). Not all bacterium contained mutations indicating that the putative T_0_ transgenic tobacco line was heterozygous.

To directly screen the T_0_ transgenic lines, genomic DNA was used as template for the first round of PCR using primers CRTISO-F/CRTISO-R (Tm= 53 °C, 30 cycles) (Fig. 3b, top panel) and the same amounts of amplification products (1.2 ng) used as templates in a second PCR using primers CRTISO-T/CRTISO-R (Tm = 62 °C, 20 cycles) (Fig. 3b, bottom panel). The absence of amplification indicated the presence of mutations in both alleles, although this method cannot distinguish between homozygous and biallelic mutations. The second PCR analysis of all 39 T_0_ plants were shown in Additional file 5: Fig. 3B, and further analyzed by sequencing the primary amplification products (primers CRTISO-F/CRTISO-R). Sequence analysis identified the three plants previously identified by MSBSP-PCR to be homozygous mutants (Additional file 6: Fig. 4A and 4B) while the remaining 13 plants contained heterozygous mutations (Additional file 6: Fig. 4C).

**Fig. 4:**
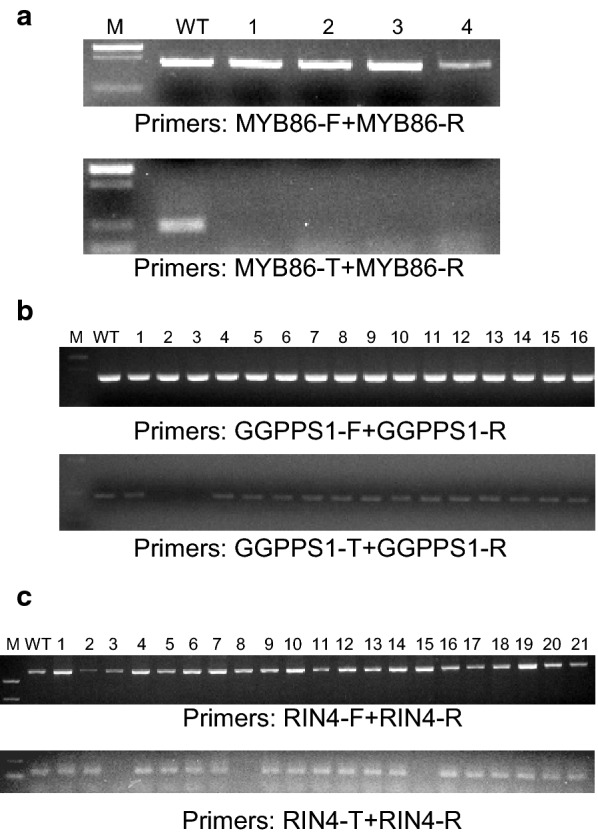
Identification of CRISPR/Cas9-induced mutants in tobacco by MSBSP-PCR. **a** Identification of *MYB86* mutants. M, marker; L1-4 are homozygous/biallelic *MYB86* mutant plants. **b** Identification of *GGPPS1* mutants. M, marker; samples #2 and 3 are homozygous/biallelic mutant plants. **c** Identification of *RIN4* mutants. M, marker; samples #3, 8 and 15 are homozygous/biallelic *RIN4* mutant plants

To further test the accuracy of our method, we performed CRISPR/Cas9-mediated mutagenesis of three additional tobacco genes, *NtMYB86*, *NtRIN4* and *NtGGPPS1*, (GenBank accession numbers XM_016625541.1, XM_009803000.1, XM_016593708.1 respectively) and analyzed the presence of mutations by MSBSP-PCR. The primers of CRISPR/Cas9 constructs were designed to target each of the three genes (Additional file 2: Table 1) and transgenic plants produced via *Agrobacterium*-mediated transformation. Putative T_0_ transgenic tobacco plants for all genes were used to extract genomic DNA and screen for the presence of mutations. For *NtMYB86* (*Nicotiana tabacum* transcription factor MYB86), a primary PCR was performed with primers MYB86-F/MYB86-R (Tm = 54 °C, 30 cycles) (Fig. 4a, upper panel) and the products used for the secondary amplification using primers MYB86-T/MYB86-R (Tm = 62 °C, 30 cycles) (Fig. 4a, lower panel). The absence of amplification in the secondary PCR indicated the presence of homozygous/biallelic mutations in the examined plants (Fig. 4a, lower panel, lanes 1–4). Out of 24 T_0_ lines analyzed, three were identified as homozygous/biallelic (Additional file 7: Fig. 5) and confirmed by sequencing the products of the primary amplification (Additional file 8: Fig. 6). In the case of *NtGGPPS1* (*Nicotiana tabacum* geranylgeranyl diphosphate synthase 1), the primary PCR was performed using primers GGPPS1-F/GGPPS1-R (Tm = 55 °C, 20 cycles) (Fig. 4b, upper panel), the second PCR using primers GGPPS1-T/GGPPS1-R (Tm = 62 °C, 20 cycles) (Fig. 4b, lower panel). Lines containing homozygous/biallelic mutations were identified by the absence of secondary amplification and verified by sequencing the primary amplification products (Fig. 4b, lower panel, lanes 2–3). Out of 23 T_0_ lines analyzed, three were identified as homozygous/biallelic (Additional file 9: Fig. 7) and confirmed by sequencing the products of the primary amplification (Additional file 10: Fig. 8). These three homozygous/biallelic lines have the same mutation (Additional file 10: Fig. 8), an unusual case in CRISPR/Cas9-induced mutations and it might indicate that the three T_0_ transgenic plants were differentiated from the same callus. Finally, T_0_ plants containing CRISPR/Cas9 constructs targeting *NtRIN4* (*Nicotiana tabacum* RPM1-interacting protein 4) were analyzed by performing the primary PCR with primers RIN4-F/RIN4-R (Tm = 55 °C, 20 cycles) (Fig. 4c, upper panel) and the secondary PCR using primers RIN4-T/RIN4-R (Tm = 63 °C, 25 cycles) (Fig. 4c, lower panel). A total of three lines containing homozygous/biallelic mutations were detected (Fig. 4c, lower panel, lanes 3, 8 and 15 and Additional file 11: Fig. 9, lanes 5, 7 and 9) and confirmed by sequencing of the primary PCR products (Additional file 12: Fig. 10).

**Fig. 5:**
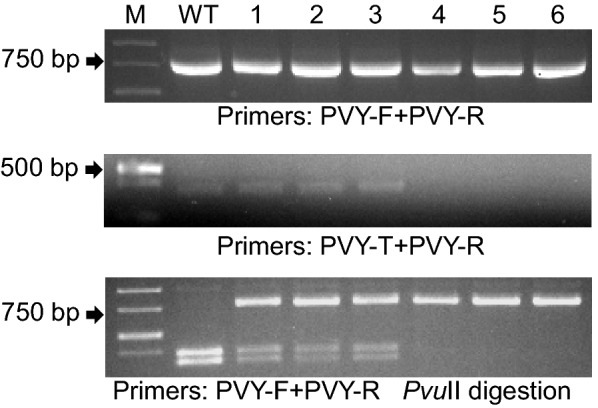
Identification of the CRISPR/Cas9-induced mutants in T_1_ tobacco plants by MSBSP-PCR. A first PCR reaction was conducted with genomic DNA isolated from T_1_ transgenic plants using locus primers PVY-F and PVY-R (upper panel) and the amplification products used as template for a secondary PCR with the target specific primer PVY-T and PVY-R (middle panel). Lanes 4-6 were identified as homozygous/biallelic mutants based on the absence of amplification product. To confirm the results of the MSBSP-PCR, the products of the first PCR were digested with *Pvu*II (lower panel). Complete digestion of the amplification product indicates a WT gene sequence (WT lane); lanes 1-3 show a combination of digested and undigested product indicating that the plants were heterozygous. The undigested products in lines 4-6 identify the plants as homozygous or biallelic mutants

Aside from the detection of mutations in the T_0_ generation, it is important to have a quick screening procedure to analyze the T_1_ progeny. For this purpose we analyzed the segregating population of a T_0_ transformant containing a CRISPR/Cas9 cassette targeting the *NtPVY* gene (Eukaryotic translation initiation factor eIF4E−1, GenBank accession number XM_009769718.1). Genomic DNA was isolated from a number of T_1_ individuals; the initial PCR was performed using primers PVY-F/PVY-R (Tm = 53 °C, 30 cycles) (Fig. 5, upper panel) and the amplification products subjected to a secondary PCR using primers PVY-T/PVY-R (Tm = 65 °C, 16 cycles) (Fig. 5, middle panel). The absence of amplification products in samples 4, 5 and 6 indicated the presence of homozygous or biallelic mutations (Fig. 5, middle panel). The target site for the CRISPR/Cas9 construct used for this gene contained a *Pvu*II cleavage site at the predicted DSB position in order to detect the presence of mutations by the destruction of the restriction site. When the products from the primary amplification were digested with *Pvu*II, the WT sample showed complete digestion of the amplicon, while samples 1-3 showed a composite pattern of digested and undigested product, identifying these plants as putative heterozygous (Fig. 5, lower panel). Consistent with the results of the MSBSP-PCR analysis, samples 4-6 were not cut by *Pvu*II confirming the presence of either homozygous or biallelic mutations. Analysis of 33 T_1_ plants by MSBSP-PCR identified 3 putative homozygous/biallelic mutants (Additional file 13: Fig. 11), and DNA sequencing further confirmed that all 3 T_1_ plants contained homozygous mutations (Additional file 14: Fig. 12).

**Fig. 6:**
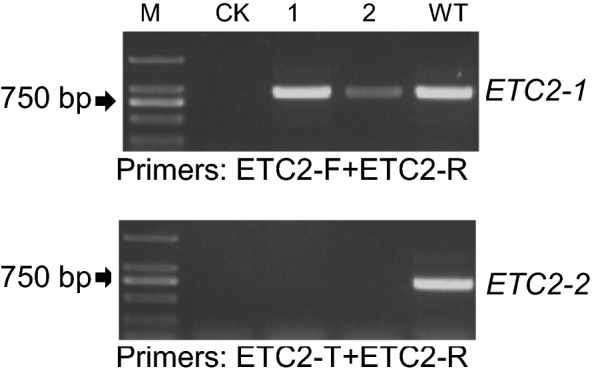
Identification of CRISPR/Cas9-induced *etc2* mutants in *Arabidopsis* by MSBSP-PCR. Genomic DNA was amplified by PCR using the locus primers ETC2-F and ETC2-R (upper panel) and the amplification products used as template for a second PCR using he target specific primer ETC2-T and the locus primer ETC2-R (lower panel). M, marker; CK, negative control with ddH_2_O as template. L1 and L2, homozygous *etc2* mutant plants; WT, wild type

To test the effectiveness of MSBSP-PCR to screen mutants in additional plant species, we obtained a previously characterized homozygous *Arabidopsis* mutant produced by CRISPR/Cas9 targeting the *AtETC2* gene (Enhancer of TRY and CPC 2; GENE ID AT2G30420) [31]. Wild type and homozygous mutant plants were grown and primary PCR of the genomic DNA performed using primers ETC2-F/ETC2-R (Tm = 55 °C, 30 cycles) (Fig. 6, upper panel). Products from the primary PCR were used as templates for the secondary PCR using ETC2-T/ETC2-R as primers (Tm = 65 °C, 23 cycles) (Fig. 6, lower panel). As in the case of tobacco, homozygous mutants were identified by the absence of amplification in the secondary PCR (Fig. 6, lower panel lanes 1–2).

## Discussion

In this work, we describe the development of MSBSP-PCR, a new method to identify mutants generated by the CRISPR/Cas9 system and prove its efficiency in tobacco and *Arabidopsis*. A number of methods are already available to detect CRISPR/Cas9-induced mutations [12–28], all of which have advantages and disadvantages. The main advantages of our method are its technical simplicity, quickness and low cost, it could be used to screen homozygous/biallelic mutants from T_0_ plants, or the descendants of heterozygous/monoalleic mutants. Moreover, the two-round of PCR enhanced the accuracy and reproducibility of the PCR results. In high complexity genomes such as those of polyploid species, like cotton, tobacco, wheat, etc., PCR amplifications directly with primer set of primer-T and primer-R and genomic DNA as templates may cause many nonspecific amplification products. On the other hand, the method cannot distinguish between homozygous and biallelic mutations and it is therefore important to sequence the target site once the mutated plants have been identified. It is also critical to carefully determine the stringency conditions in the secondary PCR in order to distinguish between WT and mutated templates. The MSBSP-PCR method is only useful to detect mutations close and upstream of the PAM, which are the most frequent when using the CRISPR/Cas9 system. Mutations far from the PAM will require some kind of preliminary characterization by PCR amplification of the targeted genomic fragment followed by TA cloning and sequencing of multiple recombinant clones, thus allowing us to design a proper target primer.

Moreover, the numbers of cycles in second PCR to screen CRISPR/Cas9-induced mutants of *NtCRTISO*, *NtMYB86*, *NtRIN4* and *NtGGPPS1* are distinctive, the proper number of cycles is determined by PCR parameters, including Tm, concentration of template and amplification efficiency of primers. Generally, 23 cycles is used for preliminary experiment. If the second PCR obtains large amount of product with both WT and transgenic lines as templates, the number of cycles can be reduced; while the second PCR obtains little amount of product in WT lines, the number of cycles must be increased.

## Conclusion

We have developed a fast, cheap and easy screening method for CRISPR/Cas9 system-induced homozygous/biallelic mutant identification. This method can be used to screen CRISPR/Cas9 system-induced mutant in tobacco and *Arabidopsis*.

## Additional files

**Additional file 1: **Sequences of each gene and CRISPR/Cas9-induced mutants or synthesized templates used in all experiments.

**Additional file 2: Table S1.** List of primers used in this study.

**Additional file 3: Fig. 1.** The yield of temperature gradient PCR with different single point mutation templates of *NtCRTISO* (synthesized) and different combination of primers was detected by agarose gel electrophoresis.

**Additional file 4: Fig. 2.** The yield of temperature gradient PCR with different multiple point mutation templates of *NtCRTISO* (synthesized) and different combination of primers was detected by agarose gel electrophoresis.

**Additional file 5: Fig. 3.** Identification of CRISPR/Cas9-induced *crtiso* mutants in tobacco by MSBSP-PCR.

**Additional file 6: Fig. 4.** The sequencing and sequences analysis of different clones of *NtCRTISO* transgenic lines.

**Additional file 7: Fig. 5.** Identification of CRISPR/Cas9-induced *myb86* mutants in tobacco by MSBSP-PCR.

**Additional file 8: Fig. 6.** The sequencing and sequences analysis of different transgenic lines of *NtMYB86.*

**Additional file 9: Fig. 7.** Identification of CRISPR/Cas9-induced *ggpps1* mutants in tobacco by MSBSP-PCR.

**Additional file 10: Fig. 8.** The sequencing and sequences analysis of different transgenic lines of *NtGGPPS1.*

**Additional file 11: Fig. 9.** Identification of CRISPR/Cas9-induced *rin4* mutants in tobacco by MSBSP-PCR.

**Additional file 12: Fig. 10.** The sequencing and sequences analysis of different transgenic lines of *NtRIN4.*

**Additional file 13: Fig. 11.** Identification of CRISPR/Cas9-induced *pvy* mutants in tobacco by MSBSP-PCR.

**Additional file 14: Fig. 12.** The sequencing and sequences analysis of different transgenic lines of *NtPVY.*

## Abbreviations

MSBSP-PCR: mutation sites based specific primers polymerase chain reaction
SSN: ssequence-specific nucleases
ZFNs: zinc-finger nucleases
CRISPR: Clustered Regularly Interspaced Short Palindromic Repeats
Cas9: CRISPR-associated protein 9
DSBs: double-strand breaks
NHEJ: non-homologous end joining
sgRNA: single-guide RNA
PAM: protospacer-adjacent motif
HRM: high-resolution melting
ACT-PCR: annealing at critical temperature PCR
Tm: melting temperature

## References

[1] Bibikova M, Beumer K, Trautman JK, Carroll D (2003). Enhancing gene targeting with designed zinc finger nucleases. Science.

[2] Porteus MH, Baltimore D (2003). Chimeric nucleases stimulate gene targeting in human cells. Science.

[3] Li T, Huang S, Jiang WZ, Wright D, Spalding MH, Weeks DP (2011). TAL nucleases (TALNs): hybrid proteins composed of TAL effectors and *Fok*I DNA-cleavage domain. Nucleic Acids Res.

[4] Cho SW, Kim S, Kim JM, Kim JS (2013). Targeted genome engineering in human cells with the Cas9 RNA-guided endonuclease. Nat Biotechnol.

[5] Mali P, Yang L, Esvelt KM, Aach J, Guell M, DiCarlo JE (2013). RNA-guided human genome engineering via Cas9. Science.

[6] Hwang WY, Fu Y, Reyon D, Maeder ML, Tsai SQ, Sander JD (2013). Efficient genome editing in zebrafish using a CRISPR-Cas system. Nat Biotechnol.

[7] Boch J, Scholze H, Schornack S, Landgraf A, Hahn S, Kay S (2009). Breaking the code of DNA binding specificity of TAL-type III effectors. Science.

[8] Moscou MJ, Bogdanove AJ (2009). A simple cipher governs DNA recognition by TAL effectors. Science.

[9] Wood AJ, Lo TW, Zeitler B, Pickle CS, Ralston EJ, Lee AH (2011). Targeted genome editing across species using ZFNs and TALENs. Science.

[10] Joung JK, Sander JD (2013). TALENs: a widely applicable technology for targeted genome editing. Nat Rev Mol Cell Biol.

[11] Yi P, Li W, Ou G (2014). The application of transcription activator-like effector nucleases for genome editing in *C. elegans*. Methods.

[12] Urnov FD, Rebar EJ, Holmes MC, Zhang HS, Gregory PD (2010). Genome editing with engineered zinc finger nucleases. Nat Rev Genet.

[13] Sander JD, Dahlborg EJ, Goodwin MJ, Cade L, Zhang F, Cifuentes D (2011). Selection-free zinc-finger-nuclease engineering by context-dependent assembly (CoDA). Nat Methods.

[14] Zhang H, Gou F, Zhang J, Liu W, Li Q, Mao Y (2016). TALEN-mediated targeted mutagenesis produces a large variety of heritable mutations in rice. Plant Biotechnol J.

[15] Cong L, Ran FA, Cox D, Lin S, Barretto R, Habib N (2013). Multiplex genome engineering using CRISPR/Cas systems. Science.

[16] Zhang H, Zhang J, Wei P, Zhang B, Gou F, Feng Z (2014). The CRISPR/Cas9 system produces specific and homozygous targeted gene editing in rice in one generation. Plant Biotechnol J.

[17] Wang S, Zhang S, Wang W, Xiong X, Meng F, Cui X (2015). Efficient targeted mutagenesis in potato by the CRISPR/Cas9 system. Plant Cell Rep.

[18] Iqbal Z, Sattar MN, Shafiq M (2016). CRISPR/Cas9: a tool to circumscribe cotton leaf curl disease. Front Plant Sci..

[19] Zhang Y, Liang Z, Zong Y, Wang Y, Liu J, Chen K (2016). Efficient and transgene-free genome editing in wheat through transient expression of CRISPR/Cas9 DNA or RNA. Nat Commun..

[20] Gao W, Long L, Tian X, Xu F, Liu J, Singh PK (2017). Genome editing in cotton with the CRISPR/Cas9 system. Front Plant Sci..

[21] Mao Y, Botella JR, Zhu JK (2017). Heritability of CRISPR/Cas9-targeted gene modifications in plants. Cell Mol Life Sci.

[22] Montgomery J, Wittwer CT, Palais R, Zhou L (2007). Simultaneous mutation scanning and genotyping by high-resolution DNA melting analysis. Nat Protoc.

[23] Shan Q, Wang Y, Li J, Zhang Y, Chen K, Liang Z (2013). Targeted genome modification of crop plants using a CRISPR-Cas system. Nat Biotechnol.

[24] Thomas HR, Percival SM, Yoder BK, Parant JM (2014). High-throughput genome editing and phenotyping facilitated by high resolution melting curve analysis. PLoS ONE.

[25] Zhu X, Xu Y, Yu S, Lu L, Ding M, Cheng J (2014). An efficient genotyping method for genome-modified animals and human cells generated with CRISPR/Cas9 system. Sci Rep..

[26] Liu WS, Zhu XH, Lei MG, Xia QY, Botella JR, Zhu JK (2015). A detailed procedure for CRISPR/Cas9-mediated gene editing in *Arabidopsis thaliana*. Sci Bull..

[27] Ramlee MK, Yan T, Cheung AM, Chuah CT, Li S (2015). High-throughput genotyping of CRISPR/Cas9-mediated mutants using fluorescent PCR-capillary gel electrophoresis. Sci Rep..

[28] Hua Y, Wang C, Huang J, Wang K (2017). A simple and efficient method for CRISPR/Cas9-induced mutant screening. J Genet Genom..

[29] Murashige T, Skoog F (1962). A revised medium for rapid growth and bio assays with tobacco tissue cultures. Physiol Plant.

[30] Shi Y, Guo J, Zhang W, Jin L, Liu P, Chen X (2015). Cloning of the lycopene β-cyclase gene in *Nicotiana tabacum* and its overexpression confers salt and drought tolerance. Int J Mol Sci.

[31] Wang ZP, Xing HL, Dong L, Zhang HY, Han CY, Wang XC (2015). Egg cell-specific promoter-controlled CRISPR/Cas9 efficiently generates homozygous mutants for multiple target genes in *Arabidopsis* in a single generation. Genome Biol.

