# Supplementary material for: A simple and cost-effective method for screening of CRISPR/Cas9-induced homozygous/biallelic mutants
Source: Plant Methods. 2018 May 29;14:40. doi: 10.1186/s13007-018-0305-8 (PMC5972395; doi:10.1186/s13007-018-0305-8)
Supplement: Supplementary file 1 — Additional file 1: Sequences of each gene and CRISPR/Cas9-induced mutants or synthesized templates used in all experiments. [file 13007_2018_305_MOESM1_ESM.docx]

**Additional file 1. Sequences of each gene and CRISPR/Cas9-induced mutants or** **synthesized templates used in all experiments.**

The sequence of synthesized template of *NtCRTISO*.

GTATAGGTGGGCTATGTTGTGGTGGACTTCTTGCTAGGTATGGCCAAGATGTTTTAGTACTCGAAAGCCATGATGTAGCTGGGGGTGCAGCTCACTCTTTTGATATTAAAGGGTACAAATTTGACTCTGGTCCATCATTGTTCTCCGGTTTTCAATCAAGAGGTCCTCAGGCAAATCCATTAGCACAGGTTACACTCTATCTGCTTACAATTTTGCTTTGTTTGTTTAGAATGTAAATGAATTCAAAGATAAGGAAAAAAAGGTTCAGCAGTTGGTGTATTTGAGACAAAGATACTAGCATTATTTCCCCTTTTCATACCTAGACCTTCCTTCCAAATGTTGCATTCGTCTTATGTGGATAGAACACCTCTTTAGGGCTTTATATATTAGCATTTTCTTTGTCACATCGACATTCTT

The green marker base pairs is the sequence reverse (R) primer, the blue marker base pairs is the sequence target (T) primer and the yellow marker base pairs is the PAM (TGG). The mutation sites of synthesized templates are upstream of the PAM, D1A (GGTGGACTTCTTGCTAGGT*), D12 (GGTGGACTTCTTGCTAGG**), D123 (GGTGGACTTCTTGCTAG***), D2 (GGTGGACTTCTTGCTAGG*A), D3 (GGTGGACTTCTTGCTAG*TA), D34 (GGTGGACTTCTTGCTA**TA), D456 (GGTGGACTTCTTGC***GTA), D5 (GGTGGACTTCTTGCT*GGTA), D56 (GGTGGACTTCTTGC**GGTA), D6 (GGTGGACTTCTTGC*AGGTA), D7 (GGTGGACTTCTTG*TAGGTA) and D8 (GGTGGACTTCTT*CTAGGTA).

The positions of primers and mutation site in the sequence of *NtCRTISO*.

ATTGTTCTCTTCATGATAACTGGTGATGTATCTCATTGTTAATCCATGAAATATGGACCTCAGCGTAAACTGACATTCCAGCGAAGGAAAAACATGTACTTCTGAAGTAATACTATGAGAGGATCAGCTTGTGTAGACTTCTAATGCAGATAAGTTGTCTCTCTCTACAGGAACATGTTTTTTTGACTGCAAGAATTATGTGCAGGCAAGCCAGAAGCAGATATCATTGTTATTGGAAGCGGTATAGGTGGGCTATGCTGTGGTGGACTTCTTGCTAGGTATGGCCAAGATGTTTTAGTACTCGAAAGCCATGATGTAGCTGGGGGTGCAGCTCACTCTTTTGATATTAAAGGGTACAAATTTGACTCTGGTCCATCATTGTTCTCCGGTTTTCAATCAAGAGGTCCTCAGGCAAATCCATTAGCACAGGTTACACTCT

The red marker base pairs is the sequence forward (F) primer, the green marker base pairs is the sequence reverse (R) primer, the blue marker base pairs is the sequence target (T) primer and the yellow marker base pairs is the PAM. The mutation sites were found by sequencing and marked with *, GGTGGACTTCTTGCTAG*TA, or marked with red, GGTGGACTTCTTGCTAGTGTA, GGTGGACTTCTTGGTAG*TA.

The positions of primers and mutation site in the sequence of *NtMYB86*.

CATGATATGGTATCTGATCATCAGTTCAATTGTAGCCTTTCACCCATGTCGAATTCTATTCTCACTACATCCCCGTTGGCACGTATAAAGTCGCCTTCTTTAACTACTCTTCCTCCTGATAATAGTTTTAATATCAACAAGTCCCAGAATTGGGAAGCTTGTACTCTCAGCAGCAACAGTAATGGTAGCAGTAACAGTATTGAATTACAAAGCAACTGTTCATTCTTTGATAACAATGCTGCAGCTTTCGCATGGGGATCAACAACAGCACATGGTAGTGGGAAACCAGAGAGAGAAGAAATCAAATGGTCTGAGTATTTGCAAACCCCATTTTCACTAGGTTCTAACACAATCCAGA

The red marker base pairs is the sequence forward (F) primer, the green marker base pairs is the sequence reverse (R) primer, the blue marker base pairs is the sequence target (T) primer and the yellow marker base pairs is the PAM. The mutation site was found by sequencing and marked with *, CTCTCAGCAGCAACA***A, or marked with red, CTCTCAGCAGCAACAGTTA.

The positions of primers and mutation site in the sequence of *NtGGPPS1*.

ACCTGTGATCCACGAAGCAATGCGCTATTCACTTCTCGCCGGCGGCAAAAGAGTCCGACCGATGCTCTGCCTCGCCGCCTGCGAGCTCGTCGGCGGCGACCAATCCAACGCCATGCCGGCTGCTTGCGCCGTCGAGATGATCCACACTATGTCCCTCATTCACGACGATTTACCTTGTATGGATAACGACGATCTCCGCCGTGGAAAGCCGACGAACCACAAAGTCTACGGCGAGGACGTGGCGGTCCTCGCCGGAGACTCGCTCCTCGCTTTCGCCTTCGAGTACATCGCCACCGCTACCGCCGGAGTTTCACCGTCGAGGATCCTCGCCGCCATCGGCGAACTGGCGAAAT

The red marker base pairs is the sequence forward (F) primer, the green marker base pairs is the sequence reverse (R) primer, the blue marker base pairs is the sequence target (T) primer and the yellow marker base pairs is the PAM. The mutation site was found by sequencing and marked with *, CACGACGATTTACCT*GTA.

The positions of primers and mutation site in the sequence of *NtRIN4*.

TTGGTACTCTTTCTAGCAGCCTGATAGAGGAGCCGCAGTTCCAAGATTCGGGGAGTGGGATGAGAATGATCCTCAATCTGCTGATAACTACACTCACATTTTTAACAAGGTTCGGGAGGAAAGACAATTGGGTACTGGAAATCCATCAGGGACACCAAGTAGAACATCTTACAACCCACAAAGGCAAGAAGAAAAGCAGATGGTAAAACACTTTCCCTCTATTGTCATGATTGATTCGTT

The red marker base pairs is the sequence forward (F) primer, the green marker base pairs is the sequence reverse (R) primer, the blue marker base pairs is the sequence target (T) primer and the yellow marker base pairs is the PAM. The mutation site was found by sequencing and marked with red or *, GTTCGGGAGGAAAGACTAAT, GTTCGGGAGGAAA*****T.

The positions of primers and mutation site in the sequence of *NtPVY*.

ATGTCGTGTTGATACGGGTGCGGCAAAGATTTTGAAGAGTCCGCGCAACTTAGGATGCATGCACCTTGTTTGGTGAGTTCTTTATCAGTCTAATTTCTCAAGGCACTTGAGTTATTGTGCAACTTGGACTATGTCATGCCTATTTTGATATTCTGCATCTTGGATTAGATGTTTTCAAATGCTATTATCCTGTTAGCTTTTGATGAAATCCTTGAACCATGTTGCTTAAATTCTGCAAACAGTGTTTACAATAATATCAACCACCCAAGCAAGTTAGTTGTGGGAGCAGACTTTCATTGTTTTAAGCATAAAATTGAGCCAAAGTGGGAAGATCCTGTATGTGCGAATGGAGGGAATTGGACAATGAGCTTTAGTAAGGGTAAATCTGATACCAGCTGGCTATACACGGTATGCTGAGGATATTTTAATCCAGTTCTTAATGTTAGGGCGCAGTCTCGTAAAGTTATTTTCCCCTTTGATATTATTTCAACTCTTATTTTCTCATTTGGGATTATTGTAGCTGCTGGCAATGATTGGACATCAATTCGATCATGGAGAGGAAATTTGTGGAGCAGTAGTTAGCGTCCGAAATAAGGGGGATAAAATAGCTTTATGGACCAAGAATGCTGCAAATGAAACAGCTCAGGTAATTTACTTTTTACCAATGAAATAGCCTATTTATATTACTCCCTTTGTTCCAATTTATGTGATGCATTTTCTTTTTTTGTCCGTCCCCAAAAGAATG

The red marker base pairs is the sequence forward (F) primer, the green marker base pairs is the sequence reverse (R) primer, the blue marker base pairs is the sequence target (T) primer with underline marker sequence (CAGCTG) is recognition site of restriction enzyme *Pvu*Ⅱ and the yellow marker base pairs is the PAM. The mutation site was found by sequencing and marked with red, TGATACCAGCTGGCTATACACGG.

The positions of primers and mutation site in the sequence of *AtETC2*.

CCTCACATCTTTTCTATATCTTGCATTCTCCAAACTCTACATTTTCAGTTTCTCTGTAAATTTATCTAATATTCTTCTATTTTCCAGTAGTTATGGATAATACCAACCGTCTTCGTCTTCGTCGCGGTCCCAGTCTTAGGCAAACTAAGTTCACTCGATCCCGATATGACTCTGAAGGTCTCTCTTCAATTCATTACTCTACACTCATCCTCCTAACTCGTCAGCGTTAAATTATCTTCTCATTTAATTTTTGTTTGGATAAAACGAAACAAAAATGCAGAAGTGAGTAGCATCGAATGGGAGTTTATCAGTATGACCGAACAAGAAGAAGATCTCATCTCTCGAATGTACAGACTTGTCGGTAATAGGTAACAAATTCTCTTCTTCTGTCATGTCCGATTCAACATATTTTAAAAACAACTAGTATTTTATAAAATTAAAAAAAAAAAAAAAAAAACTAGTATTTTATAATTTAAAAAAAATTCACATGGCGAGAGTAACAAATCAAAGCTGATAAAGTGTTATGTTATGGTAAACATGATTCTAGAGTAGTTTAGACTTTCGAACGTGTTTCGAGAAAAATGACATGCCAATATTTAAGCAATGACATGAGTATTTTACTTGTATAATTTCAAAAAAAGTTTTATGTTGTTGATAAACTGATAAAATGTATAATTAACAGAAACTAAAATTTCTTCTTTTCTTAAAGTGTGATGATTTTAAAAACAGTGAAAGGATTGAATAATATATTGGCTCTTGAATATTATTGTCTTTATATATATACATTTTAAATTTCATTTCTGCATTATATATGTTGACTCCTGAATTTTGATAAATGTTTTTTCAGAATTTTGTGCTCGTCTATAATTCAAAAACACATTATATGCTTGAATATTGATTGTCAGGTGGGATTTAATAGCAGGAA

The red marker base pairs is the sequence forward (F) primer, the green marker base pairs is the sequence reverse (R) primer, the blue marker base pairs is the sequence target (T) primer and the yellow marker base pairs is the PAM. The mutation site was found by sequencing and marked with *, CAGAAGTGAGTAGCATC*AAT.
