# Supplementary material for: A simple and cost-effective method for screening of CRISPR/Cas9-induced homozygous/biallelic mutants
Source: Plant Methods. 2018 May 29;14:40. doi: 10.1186/s13007-018-0305-8 (PMC5972395; doi:10.1186/s13007-018-0305-8)
Supplement: Supplementary file 2 — Additional file 2: Table S1. List of primers used in this study. [file 13007_2018_305_MOESM2_ESM.doc]

**Additional file 2. Table 1. A list of the primers used in all experiments.**

| Genes |  | Primers |
| --- | --- | --- |
| **MSBSP-PCR primers** |  |  |
| *NtGGPPS1* | F | 5' ACCTGTGATCCACGAAGCAATG 3' |
|  | R | 5' ATTTCGCCAGTTCGCCGATG 3' |
|  | T | 5' CACGACGATTTACCTTGTA 3' |
|  |  |  |
|  | F | 5' ATTGTTCTCTTCATGATAACTGG 3' |
| *NtCRTISO* | R | 5' AGAGTGTAACCTGTGCTAATGG 3' |
|  | T | 5 'GGTGGACTTCTTGCTAGGTA 3' |
|  |  |  |
|  | F | 5' CATGATATGGTATCTGATCATCA 3' |
| *NtMYB86* | R | 5' TCTGGATTGTGTTAGAACCTAGTG 3' |
|  | T | 5' CTCTCAGCAGCAACAGTAA 3' |
|  |  |  |
|  | F | 5' TTGGTACTCTTTCTAGCAGCCT 3' |
| *NtRIN4* | R | 5' AACGAATCAATCATGACAATAGA 3' |
|  | T | 5' GTTCGGGAGGAAAGACAAT 3' |
|  |  |  |
|  | F | 5' ATGTCGTGTTGATACGGGTG 3' |
| *NtPVY* | R | 5' CATTCTTTTGGGGACGGACAAA 3' |
|  | T | 5' TGATACCAGCTGGCTATACA 3' ( *Pvu* Ⅱ) |
|  |  |  |
|  | F | 5' CCTCACATCTTTTCTATATCTTGCATTC 3' |
| *AtETC2* | R | 5' TTCCTGCTATTAAATCCCACCTGAC 3' |
|  | T | 5' CAGAAGTGAGTAGCATCGAAT 3' |
|  |  |  |
|  | T | 5' GGTGGACTTCTTGCTAGGTA 3' |
|  | T1 | 5' GTGGACTTCTTGCTAGGTAT 3' |
| *NtCRTISO* | T2 | 5' TGGACTTCTTGCTAGGTATG 3' |
| (synthesized) | T3 | 5' TGGTGGACTTCTTGCTAGGT 3' |
|  | T4 | 5' GTGGTGGACTTCTTGCTAGG 3' |
|  | R | 5' AGAGTGTAACCTGTGCTAATGG 3' |
| **CRISPR/Cas9 constructs primers** |  |  |
| pHSE401-CRTISO | F | 5' GATTGGTGGACTTCTTGCTAGGTA 3' |
|  | R | 5' AAACTACCTAGCAAGAAGTCCACC 3' |
| pHSE401-PVY | F | 5' ATTGTGATACCAGCTGGCTATACA 3' |
|  | R | 5' AAACTGTATAGCCAGCTGGTATCA 3' |
| pHSE401-GGPPS1 | F | 5' ATTGCACGACGATTTACCTTGTA 3' |
|  | R | 5' AAACTACAAGGTAAATCGTCGTG 3' |
| pHSE401-MYB86 | F | 5' ATTGCTCTCAGCAGCAACAGTAA 3' |
|  | R | 5' AAACTTACTGTTGCTGCTGAGAG 3' |
| pHSE401-RIN4 | F | 5' ATTGGTTCGGGAGGAAAGACAAT 3' |
|  | R | 5' AAACATTGTCTTTCCTCCCGAAC 3' |

Blue marked sequences were the adaptor of vectors.
