# Supplementary material for: A simple and cost-effective method for screening of CRISPR/Cas9-induced homozygous/biallelic mutants
Source: Plant Methods. 2018 May 29;14:40. doi: 10.1186/s13007-018-0305-8 (PMC5972395; doi:10.1186/s13007-018-0305-8)
Supplement: Supplementary file 4 — Additional file 4: Fig. 2. The yield of temperature gradient PCR with different multiple point mutation templates of NtCRTISO (synthesized) and different combination of primers was detected by agarose gel electrophoresis. [file 13007_2018_305_MOESM4_ESM.pdf]

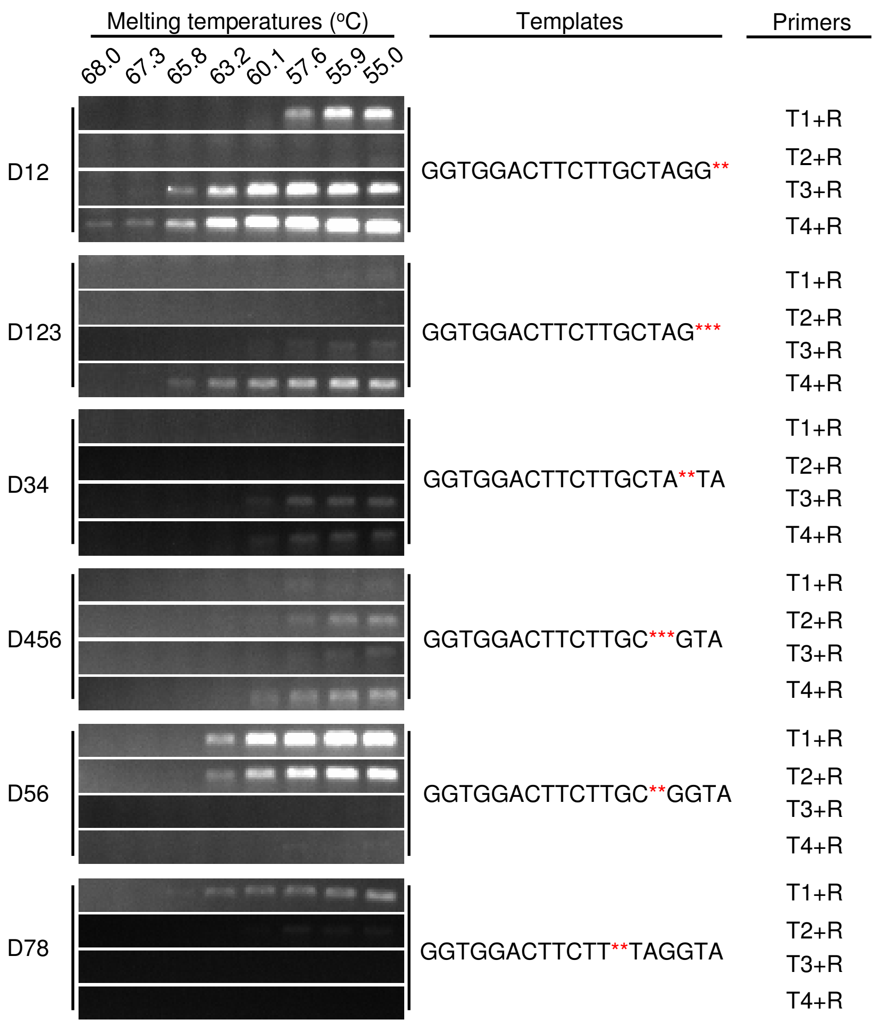


Supplementary Figure 2. The yield of temperature gradient PCR with different multiple point mutation templates of *NtCRTISO* (synthesized) and different combination of primers was detected by agarose gel electrophoresis. Equal amount of each templates was added in each PCR system. The red asterisk indicates a lack of a base pair. At least three independent biological replications were amplified to each PCR.
