# Supplementary material for: A simple and cost-effective method for screening of CRISPR/Cas9-induced homozygous/biallelic mutants
Source: Plant Methods. 2018 May 29;14:40. doi: 10.1186/s13007-018-0305-8 (PMC5972395; doi:10.1186/s13007-018-0305-8)
Supplement: Supplementary file 5 — Additional file 5: Fig. 3. Identification of CRISPR/Cas9-induced crtiso mutants in tobacco by MSBSP-PCR. [file 13007_2018_305_MOESM5_ESM.pdf]

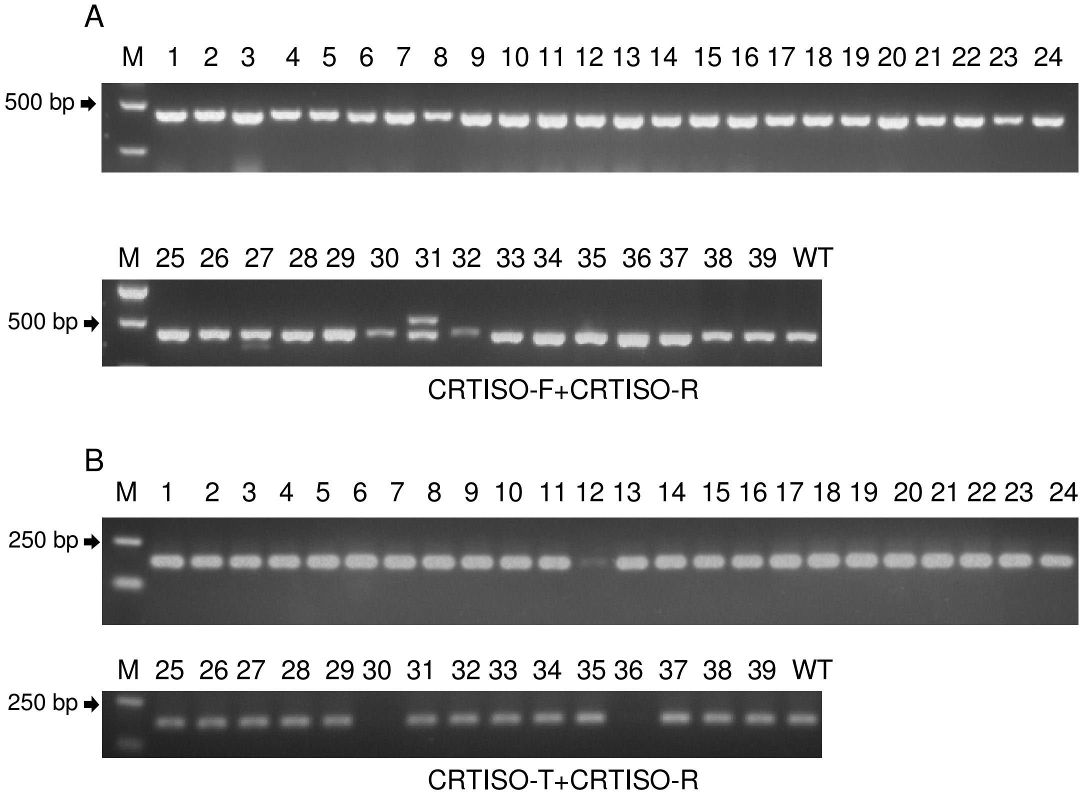

Supplementary Figure 3. Identification of CRISPR/Cas9-induced *crtiso* mutants in tobacco by MSBSP-PCR. A, the first PCR with CRTISO-F+CRTISO-R as primers and DNA of 39 T_0 plants as template. Equal amount of each DNA of 39 T_0 plants (40 ng) was added in each PCR system. B, the second PCR with CRTISO-T+CRTISO-R as primers and the products of the first PCR were added as templates. At least three independent biological replications were amplified to each PCR.
