# Supplementary material for: A simple and cost-effective method for screening of CRISPR/Cas9-induced homozygous/biallelic mutants
Source: Plant Methods. 2018 May 29;14:40. doi: 10.1186/s13007-018-0305-8 (PMC5972395; doi:10.1186/s13007-018-0305-8)
Supplement: Supplementary file 6 — Additional file 6: Fig. 4. The sequencing and sequences analysis of different clones of NtCRTISO transgenic lines. [file 13007_2018_305_MOESM6_ESM.pdf]

A

WT	5′	GGTGGACTTCTTGCTAGGTATGG	3′
L12	5′	GGTGGACTTCTTGCTAG*TATGG	3′
L30	5′	GGTGGACTTCTTGCTAGTGTATGG	3′
L36	5′	GGTGGACTTCTTG****GTATGG	3′

B

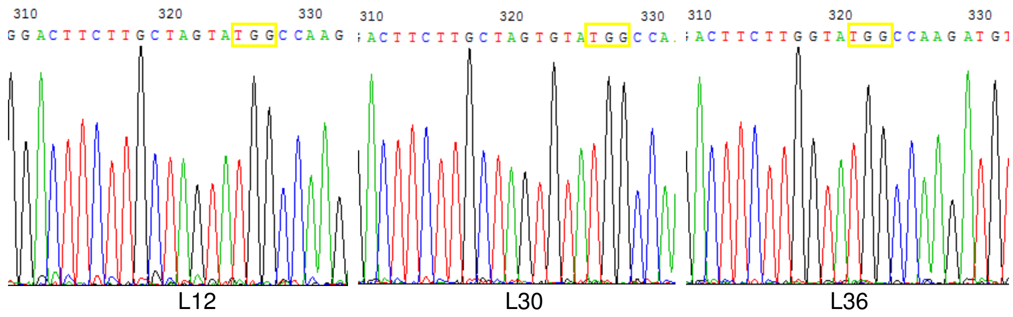

C

WT	5′	GGTGGACTTCTTGCTAGGTATGGCCAAGATGTTTTAGT	3′
L1	5′	GGTGGACTTCTTGCTA*GTATGGCCAAGATGTTTTAGT	3′
L2	5′	GGTGGACTTCTTGCT**********ATGATGT******	3′
L5	5′	GGTGGACTTCTTGCTA*GTATGGCCAAGATGTTTTAGT	3′
L8	5′	GGTGGACTTCTTGCTGG******CCAAGATGTTTTAGT	3′
L12	5′	GGTGGACTTCTTGCTAG*TATGGCCAAGATGTTTTAGT	3′
L15	5′	GGTGGACTTCTTGCTAGTGGTATGGCCAAGATGTTTTAGT	3′
L18	5′	GGTGGACTTCTTGCTA*GTATGGCCAAGATGTTTTAGT	3′
L21	5′	GGTGGACTTCTTGCTGG******CCAAGATGTTTTAGT	3′
L24	5′	GGTGGACTTCGTA********TGGCCAAGATGTTTTAGT	3′
L27	5′	* a fragment deletion of 82 bp in all *AGATGTTTTAGT	3′
L29	5′	GGTGGACTTCTTGCTAGTGGTATGGCCAAGATGTTTTAGT	3′
L30	5′	GGTGGACTTCTTGCTAGTGTATGGCCAAGATGTTTTAGT	3′
L36	5′	GGTGGACTTCTTG****GTATGGCCAAGATGTTTTAGT	3′

Supplementary Figure 4. The sequencing and sequences analysis of different clones of *NtCRTISO* transgenic lines. L12, L30 and L36 of *CRTISO* are homozygous. M13 was the sequencing primer; the sequences of wild type *CRTISO* and homozygous transgenic mutant lines (A) and heterozygous mutations (C), the blue marked TGG was the PAM, the red marked was the insertion/mismatched base pair and the * was the deleted base pair; sequencing chromatograms for L12, L30 and L36 clones (B). The yellow boxes marked sequences was the PAM (TGG). At least twenty bacteria clones were used for sequencing to each putative transgenic plant.
