# Supplementary material for: A simple and cost-effective method for screening of CRISPR/Cas9-induced homozygous/biallelic mutants
Source: Plant Methods. 2018 May 29;14:40. doi: 10.1186/s13007-018-0305-8 (PMC5972395; doi:10.1186/s13007-018-0305-8)
Supplement: Supplementary file 7 — Additional file 7: Fig. 5. Identification of CRISPR/Cas9-induced myb86 mutants in tobacco by MSBSP-PCR. [file 13007_2018_305_MOESM7_ESM.pdf]

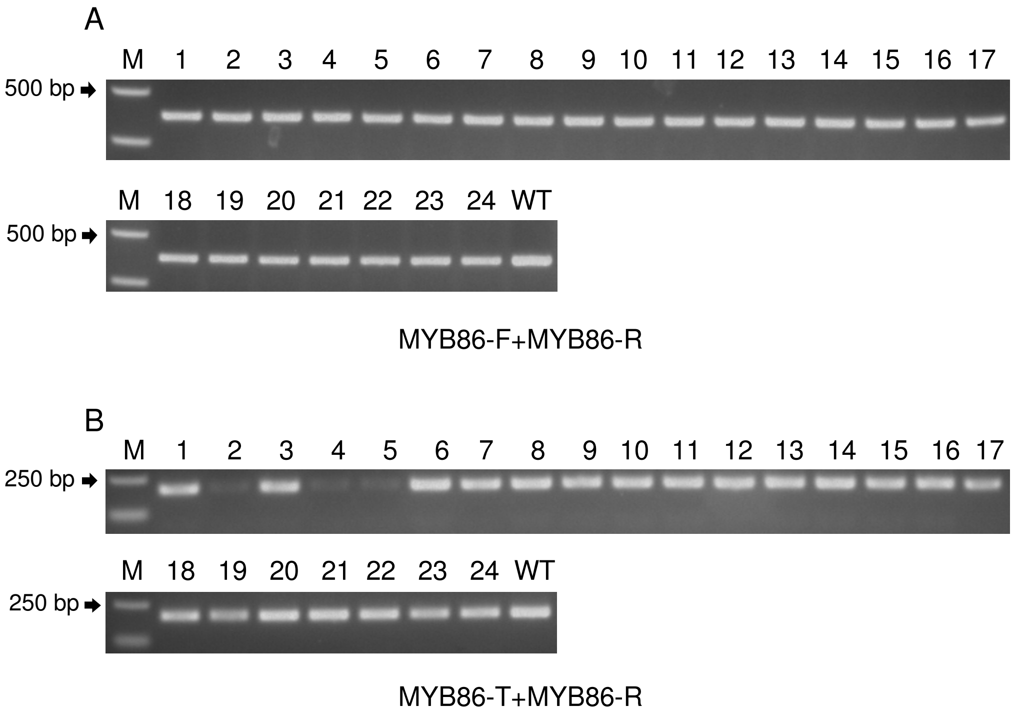

Supplementary Figure 5. Identification of CRISPR/Cas9-induced *myb86* mutants in tobacco by MSBSP-PCR. A, the first PCR with MYB86-F+MYB86-R as primers and DNA of 24 T_0 plants as template. Equal amount of each DNA of 24 T_0 plants (40 ng) was added in each PCR system. B, the second PCR with MYB86-T+MYB86-R as primers and the products of the first PCR were added as templates. At least three independent biological replications were amplified to each PCR.
