# Supplementary material for: A simple and cost-effective method for screening of CRISPR/Cas9-induced homozygous/biallelic mutants
Source: Plant Methods. 2018 May 29;14:40. doi: 10.1186/s13007-018-0305-8 (PMC5972395; doi:10.1186/s13007-018-0305-8)
Supplement: Supplementary file 8 — Additional file 8: Fig. 6. The sequencing and sequences analysis of different transgenic lines of NtMYB86. [file 13007_2018_305_MOESM8_ESM.pdf]

A

WT	5′	CTCTCAGCAGCAACAGTAATGG	3′
L2	5′	CTCTCAGCAGCAACA***ATGG	3′
L4	5′	CTCTCAGCAGCAACA***ATGG	3′
L5	5′	CTCTCAGCAGCAACAGTTAATGG	3′

B

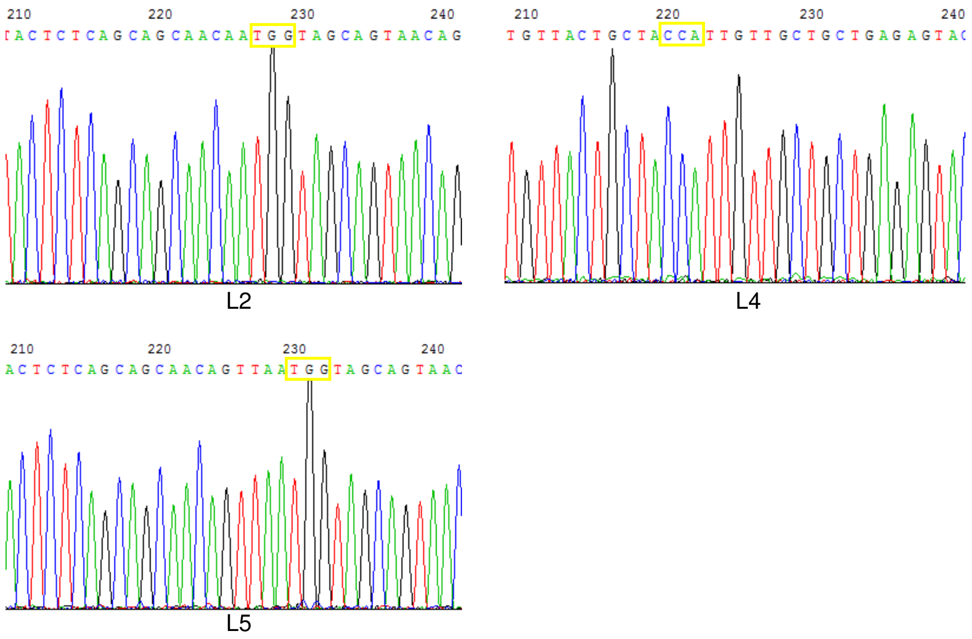

Supplementary Figure 6. The sequencing and sequences analysis of different transgenic lines of *NtMYB86*. TA clones of L2, L4 and L5 were constructed with primers of MYB86-F/MYB86-R. M13 was the sequencing primer; the sequences of wild type *MYB86* and transgenic mutant lines (A), the blue marked TGG was the PAM, the red marked was the inserted base pair and the * was the deletion sequence; sequencing chromatograms (B). The yellow boxes marked sequences was the PAM (TGG/CCA). At least twenty bacteria clones were used for sequencing to each putative transgenic plant.
