# Supplementary material for: A simple and cost-effective method for screening of CRISPR/Cas9-induced homozygous/biallelic mutants
Source: Plant Methods. 2018 May 29;14:40. doi: 10.1186/s13007-018-0305-8 (PMC5972395; doi:10.1186/s13007-018-0305-8)
Supplement: Supplementary file 10 — Additional file 10: Fig. 8. The sequencing and sequences analysis of different transgenic lines of NtGGPPS1. [file 13007_2018_305_MOESM10_ESM.pdf]

A

WT	5′	CACGACGATTTACCTTGTATGG	3′
L3	5′	CACGACGATTTACCT*GTATGG	3′
L9	5′	CACGACGATTTACCT*GTATGG	3′
L11	5′	CACGACGATTTACCT*GTATGG	3′

B

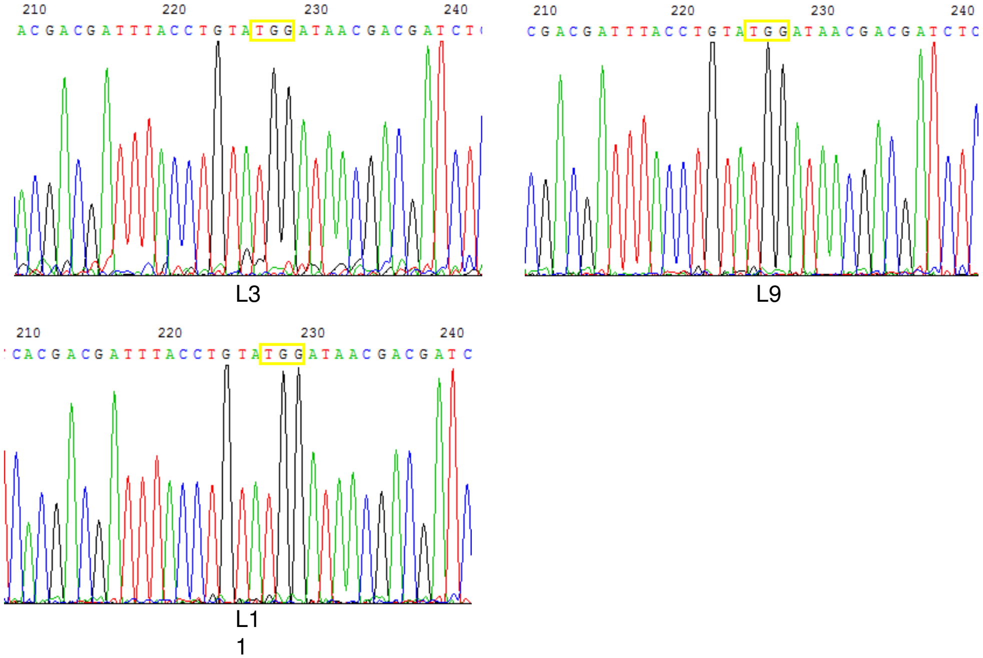

Supplementary Figure 8. The sequencing and sequences analysis of different transgenic lines of *NtGGPPS1*. TA clones of L3, L9 and L11 was constructed with primers of GGPPS1-F/GGPPS1-R. M13 was the sequencing primer; the sequences of wild type *GGPPS1* and transgenic mutant lines (A), the blue marked TGG was the PAM and the * was the deletion sequence; sequencing chromatograms (B). The yellow boxes marked sequences was the PAM (TGG). At least twenty bacteria clones were used for sequencing to each putative transgenic plant.
