# Supplementary material for: A simple and cost-effective method for screening of CRISPR/Cas9-induced homozygous/biallelic mutants
Source: Plant Methods. 2018 May 29;14:40. doi: 10.1186/s13007-018-0305-8 (PMC5972395; doi:10.1186/s13007-018-0305-8)
Supplement: Supplementary file 12 — Additional file 12: Fig. 10. The sequencing and sequences analysis of different transgenic lines of NtRIN4. [file 13007_2018_305_MOESM12_ESM.pdf]

A

WT	5′	GTTCGGGAGGAAAGACAATTGG	3′
L5	5′	GTTCGGGAGGAAAGACTAATTGG	3′
L7	5′	GTTCGGGAGGAAAGACTAATTGG	3′
L9	5′	GTTCGGGAGG*****CAATTGG	3′

B

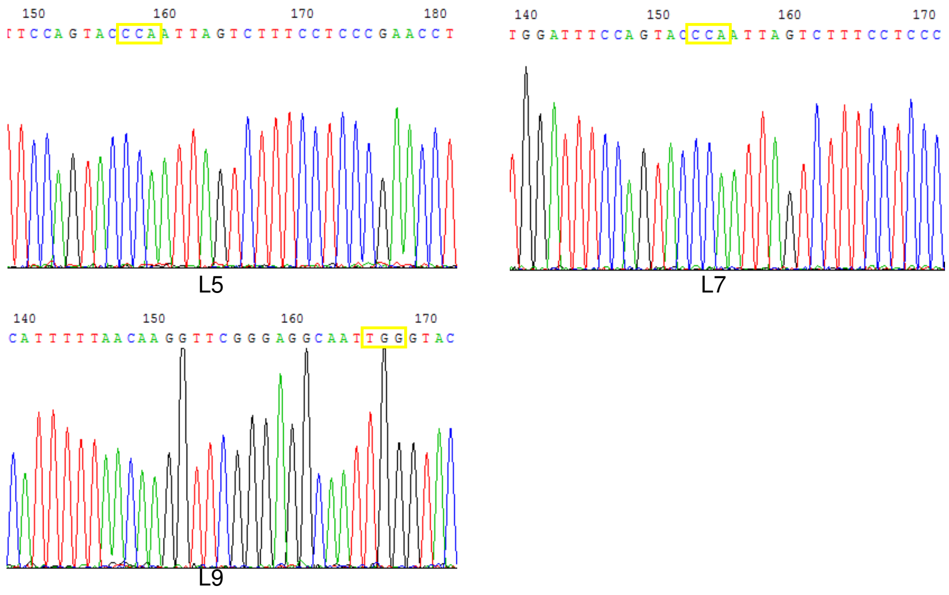

Supplementary Figure 10. The sequencing and sequences analysis of different transgenic lines of *NtRIN4*. TA clones of L5, L7 and L9 were constructed with primers of RIN4-F/RIN4-R. M13 was the sequencing primer; the sequences of wild type *RIN4* and transgenic mutant lines (A), the blue marked TGG was the PAM, the red marked was the insertion sequence and the * was the deletion sequence; sequencing chromatograms (B). The yellow boxes marked sequences was the PAM (TGG/CCA). At least twenty bacteria clones were used for sequencing to each putative transgenic plant.
