# Supplementary material for: A simple and cost-effective method for screening of CRISPR/Cas9-induced homozygous/biallelic mutants
Source: Plant Methods. 2018 May 29;14:40. doi: 10.1186/s13007-018-0305-8 (PMC5972395; doi:10.1186/s13007-018-0305-8)
Supplement: Supplementary file 13 — Additional file 13: Fig. 11. Identification of CRISPR/Cas9-induced pvy mutants in tobacco by MSBSP-PCR. [file 13007_2018_305_MOESM13_ESM.pdf]

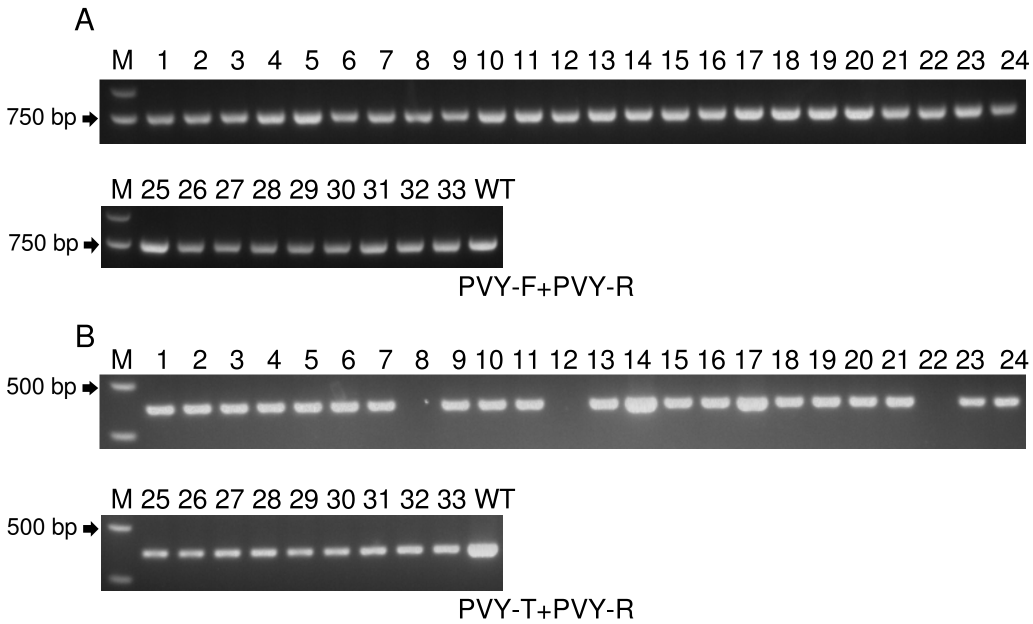

Supplementary Figure 11. Identification of CRISPR/Cas9-induced *pvy* mutants in tobacco by MSBSP-PCR. A, the first PCR with PVY-F+PVY-R as primers and DNA of 33 T_1 plants as template. Equal amount of each DNA of 33 T_1 plants (40 ng) was added in each PCR system. B, the second PCR with PVY-T+PVY-R as primers and the products of the first PCR were added as templates. At least three independent biological replications were amplified to each PCR.
