# Supplementary material for: A simple and cost-effective method for screening of CRISPR/Cas9-induced homozygous/biallelic mutants
Source: Plant Methods. 2018 May 29;14:40. doi: 10.1186/s13007-018-0305-8 (PMC5972395; doi:10.1186/s13007-018-0305-8)
Supplement: Supplementary file 14 — Additional file 14: Fig. 12. The sequencing and sequences analysis of different transgenic lines of NtPVY. [file 13007_2018_305_MOESM14_ESM.pdf]

A

WT	5′	TGATACCAGCTGGCTATACACGG	3′
L8	5′	T****************ACACGG	3′
L12	5′	T****************ACACGG	3′
L22	5′	T****************ACACGG	3′

B

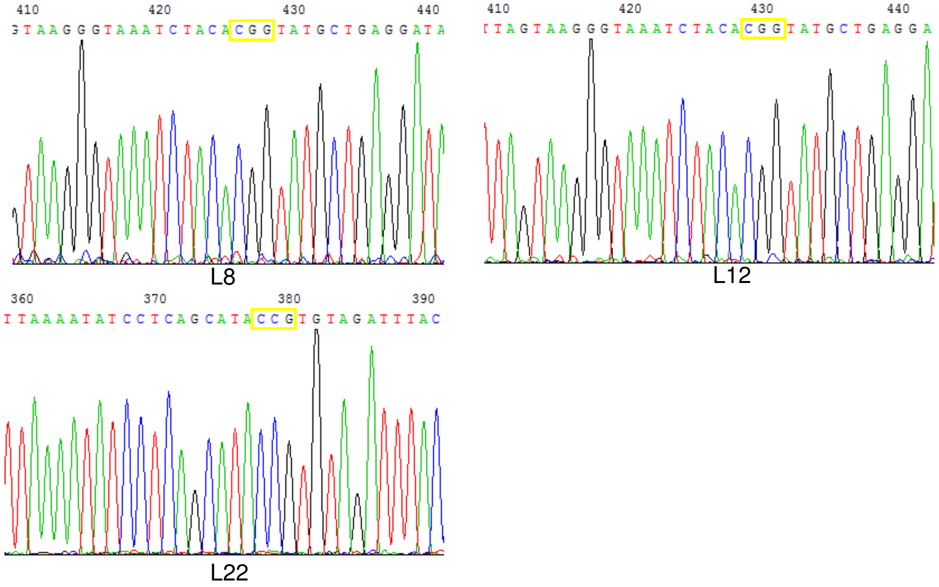

Supplementary Figure 12. The sequencing and sequences analysis of different transgenic lines of *NtPVY*. TA clones of L8, L12 and L22 were constructed with primers of PVY-F/PVY-R. M13 was the sequencing primer; the sequences of wild type *PVY* and transgenic mutant lines (A), the blue marked CGG was the PAM, the * indicated deletion sequence; sequencing chromatograms (B). The yellow boxes marked sequences was the PAM (CGG/CCG). At least twenty bacteria clones were used for sequencing to each putative transgenic plant.
